# Some Undescribed Symptoms of Angina Pectoris

**Published:** 1905-08-19

**Authors:** 


### SOME UNDESCRIBED SYMPTOMS OF ANGINA PECTORIS.

In the spring number of *Brain*, Dr. G. A. Gibson records some observations upon a patient subject to angina pectoris, which, in addition to their intrinsic interest, throw a good deal of light upon the afferent nervous mechanism of the heart by way of the sympathetic. The details of the case are as follow:—The patient, a man of 45 years, six weeks before coming under observation, had been seized with breathlessness while sitting in a train; since that date the least exertion was accompanied by dyspnœa. For five weeks he had been subject to pain in his chest, the pain being constant, though liable to exacerbations. For the first three weeks of its existence the pain remained localised in the region of the left scapula, but for the succeeding fortnight its character was changed, and a radiation of it down the left arm became established. The patient was of a robust type, but all his accessible arteries were thick and tortuous. His blood-pressure was high, measuring 160 to 170 mm. of mercury by Hill and Barnard's sphygmometer. During the anginal paroxysms the pressure appeared to be increased, but naturally measurement under the circumstances became difficult. The angina was of the variety which is relieved by amyl nitrite. There were no physical signs of organic disease of the heart, but the second sound in the aortic area was a good deal accentuated; examination with the Röntgen rays showed no evidence of aneurysm. The pain was, as has been said, constant. It was most severe over the back, and extended downwards to the level of the ninth intercostal space. Traced upward, the painful area ran up the back of the neck, and passing downward over the front of the chest, reached the level of the eighth interspace in front; it was limited below by a horizontal line at this level. It thus occupied one half of the trunk down to the eighth interspace in front, and the ninth behind, and also invaded the outer aspect of the left arm and forearm, including the thumb with the index and middle fingers. When the sensation of the affected areas came to be investigated it was discovered that while the bulk of the painful zone was intensely hyperæsthetic, the portion of it which invaded the hand was absolutely anæsthetic to all varieties of stimulus. This was the case with the whole hand-area with the exception of a small marginal zone, which was analgesic though not insensitive otherwise, and of a circular patch, the size of a threepenny-piece, situated at the junction of the thenar eminence and the palm. Over this small area sensation was perfect, a fact attributed to the function of a communicating branch of the ulnar nerve.

Up to this point the curiosity of the case, in the author's opinion, consists in the first place in the distribution of the sensory disturbance. Normally, as is well known, the line of demarcation between the sensory distributions of the ulnar and median nerves is the middle line of the ring finger; in this case it appeared to be the middle line of the middle finger. In the second place the segmental distribution was peculiar, for the inner aspect of the arm and forearm was not involved. That is to say a considerable area belonging to the segmental distributions of the seventh and eighth cervical and of the first dorsal roots was unaffected,[1] while areas above and below were deeply implicated. This is remarkable in view of the fact that the areas intact in the present case are those most frequently involved as a matter of general experience.

A further noticeable feature in the case was that the left eye was more prominent than its fellow, and the left pupil larger than the right. This condition was constant and was undoubtedly due to some irritation of the cilio-spinal fibres of the sympathetic on the left side.

Dr. Gibson considers that the real importance of the case lies in the opportunity it affords for a clear demonstration regarding the course of afferent impulses from the heart. These pass from the heart by the cardiac nerves to the cervical ganglia of the sympathetic system, enter the spinal cord by the grey rami communicantes to the posterior roots, and proceed upwards to the cortex in the ascending tracts of the cord. In the cortex they give rise to sensation, but, as Head expresses it, "the sensory and localising power of the surface of the body is enormously in excess of that of the viscera, and thus by what might be called a psychical error of judgment, the diffusion area is accepted by consciousness, and the pain is referred to the surface of the body instead of to the organ actually affected." Thus in the present case the diffusion area embraced the præcordia, shoulder and upper extremity, while the affection of the cervical sympathetic, evidenced by the pupil symptoms, proves, in Dr. Gibson's opinion, that the afferent path from the heart is contained in the sympathetic system.

[1] This statement does not tally with the segmental areas given in Head's diagrams.

